# High-Throughput Resequencing of Maize Landraces at Genomic Regions Associated with Flowering Time

**DOI:** 10.1371/journal.pone.0168910

**Published:** 2017-01-03

**Authors:** Tiffany M. Jamann, Shilpa Sood, Randall J. Wisser, James B. Holland

**Affiliations:** 1 Department of Crop Sciences, University of Illinois, Urbana, IL, United States of America; 2 Monsanto Company, 700 Chesterfield Parkway West, Chesterfield, Missouri, United States of America; 3 Department of Plant and Soil Sciences, University of Delaware, Newark, DE, United States of America; 4 USDA-ARS Plant Science Research Unit and Department of Crop and Soil Sciences, North Carolina State University, Raleigh, NC, United States of America; University of Guelph, CANADA

## Abstract

Despite the reduction in the price of sequencing, it remains expensive to sequence and assemble whole, complex genomes of multiple samples for population studies, particularly for large genomes like those of many crop species. Enrichment of target genome regions coupled with next generation sequencing is a cost-effective strategy to obtain sequence information for loci of interest across many individuals, providing a less expensive approach to evaluating sequence variation at the population scale. Here we evaluate amplicon-based enrichment coupled with semiconductor sequencing on a validation set consisting of three maize inbred lines, two hybrids and 19 landrace accessions. We report the use of a multiplexed panel of 319 PCR assays that target 20 candidate loci associated with photoperiod sensitivity in maize while requiring 25 ng or less of starting DNA per sample. Enriched regions had an average on-target sequence read depth of 105 with 98% of the sequence data mapping to the maize ‘B73’ reference and 80% of the reads mapping to the target interval. Sequence reads were aligned to B73 and 1,486 and 1,244 variants were called using SAMtools and GATK, respectively. Of the variants called by both SAMtools and GATK, 30% were not previously reported in maize. Due to the high sequence read depth, heterozygote genotypes could be called with at least 92.5% accuracy in hybrid materials using GATK. The genetic data are congruent with previous reports of high total genetic diversity and substantial population differentiation among maize landraces. In conclusion, semiconductor sequencing of highly multiplexed PCR reactions is a cost-effective strategy for resequencing targeted genomic loci in diverse maize materials.

## Introduction

The price of sequencing has dropped dramatically, and it is now cost-effective to resequence small numbers of whole genomes or obtain a large number of genome-wide markers across a large sample size using reduced-representation libraries [1–3]. As many plant genomes are large, it remains expensive to sequence whole genomes for population genetics studies, and reduced-representation sequencing strategies are preferred. Depending on the materials and the scientific question, different methods may be required, including enzyme-based reduced-representation sequencing or targeted enrichment using hybridization or PCR primers. The maize genome presents challenges, in particular, a high level of nucleotide diversity [4], extensive structural variation [5, 6] and a highly repetitive genome [7]. For this study, our aim was to discover genomic variants and genotype diverse maize inbred, hybrid, and landrace samples at specific candidate genes.

Several techniques are now available that genotype multiple single nucleotide polymorphic sites (SNPs) in a single assay, such as Sequenom or SNP arrays, but these methods require *a priori* knowledge of the polymorphisms being genotyped; a SNP discovery phase is first required [8, 9]. This strategy is unable to assay genomic variants not already captured during the discovery phase which can lead to ascertainment bias when characterizing new samples. Given knowledge of candidate regions of a genome to resequence, a targeted enrichment strategy may be used. Enrichment coupled with high-throughput sequencing can identify all genomic variants across the target space for multiple individuals that can be assayed in parallel (i.e. sequencing of libraries containing multiple samples distinguished by molecular barcodes) to obtain genotypic information from direct sequence data across a large sample.

Genotyping-by-sequencing (GBS) is now a common method used for simultaneously discovering variation and genotyping large sample sizes due to the ease of combining many samples into a single run [10]. A drawback of this approach is that there is no guarantee that a region of interest will be covered. In fact, with the low coverage sequence data commonly acquired in GBS, any one particular genome segment is likely to be missing sequence reads from most samples. This approach results in a large proportion of missing data at each nucleotide where variants are scored, because at any one site many of the individuals assayed will not have a sequenced read. For example, in a recent study in maize, the average missing data rate was 58% before imputation [11]. Genotype imputation can ameliorate some of these problems, but the effectiveness of imputation relies on linkage disequilibrium and genetic relatedness between samples with missing data and samples with known genotypes. The accuracy of imputation methods such as FiLLIN and Beagle range from 58% to 74% for diverse maize landraces, which are highly heterozygous [12]. Furthermore, low-coverage sequencing tends to represent heterozygous sites inaccurately as homozygous.

A number of different enrichment strategies can be employed to amplify target regions of the genome [13]. Most commonly, hybridization-based or PCR-based enrichment is used. Hybridization-based enrichment is an effective method to sequence non-reference genomes at regions of interest and capture information about structural variation [14]. Hybridization using oligo capture approaches have been used in crop species with success [15, 16]. However, very high-quality DNA is needed for these methods, and the repetitive nature of some genomes can be problematic [15].

PCR-based enrichment for genotyping relies on designing primers to tile across a region of interest, amplifying those regions, preparing samples for sequencing, using high-throughput sequencing to sequence samples and identifying variants. Different strategies can be employed for PCR-based enrichment, namely singleplex or multiplex PCR reactions. Massively parallel singleplex amplification using microdroplet PCR is an efficient method of sampling a large number of amplicons across a large number of samples [17]. However, this option requires specialized equipment. Another option is highly multiplexed reactions including multiple PCR primers such as Ampliseq from ThermoFisher Scientific, Inc., TruSeq amplicon panels from Illumina, or GeneRead from Qiagen. With these approaches, small amplicons are designed that tile the region of interest. These systems are easily accessible and custom panels can be easily designed; however, many of the design pipelines for highly multiplexed reactions are focused on the human genome [18]. Another advantage of these approaches is that they require only a small amount of starting DNA. Challenges to this approach include the high rate of polymorphism in the maize genome and the repetitive nature of plant genomes. We chose Ampliseq because it offers a pipeline with pre-loaded reference genomes for designing non-human panels, including for maize. Products are subsequently sequenced using semiconductor sequencing, a DNA sequencing technology based on the detection of hydrogen ions released when nucleotides are incorporated into the DNA molecule [19].

Here we demonstrate that amplicon-based enrichment coupled with semiconductor sequencing, referred to as Ampliseq, is an effective means to identify new sequence variation in multiple genomic regions across a diverse sample of maize germplasm. The objectives of this study were to validate Ampliseq in maize, create an Ampliseq panel to study candidate photoperiod response genes, develop a bioinformatics pipeline to call variants, and examine the relationship between maize races. This method offers high depth coverage of regions of interest that is suitable for population genetics studies, marker-assisted selection, or other applications where high coverage is required across a specific region(s) of interest.

## Materials and Methods

### Plant material

A panel of maize inbreds, hybrids, and landrace accessions was assembled for genotyping. A set of control inbred lines already sequenced at high coverage (B73, Mo17, CML322), and F_1_ hybrids (B73×Mo17 and B73×CML322), were included to assess the accuracy of sequence information. A sample of 19 landrace accessions from Argentina and Bolivia representing 19 named races was used to compare sequence variation in landraces to the modern inbreds (S1 Table). Tissue from five plants per accession was collected from greenhouse-grown seedlings and frozen at -80°C until tissue homogenization. Tissue homogenization was carried out in a Retch Mixer Mill MM301 (Retsch GmbH & Co., Haan, Germany) for 2 min at 25 revolutions/second. DNA was extracted with a Qiagen DNeasy kit (Qiagen, Hilden, Germany) following kit instructions.

### Ampliseq design

Target genome regions for sequencing were selected based on candidate gene information for photoperiod sensitivity in maize [20–22]. A total of 20 genome regions were selected for inclusion in the study for a total of 86,436 bp located on a total of seven chromosomes (Table 1). These regions were chosen based on genes that are known to play a role in related pathways, as well as candidate genes from genome-wide nested association mapping [20, 21]. Gene regions, as well as 2 kb upstream of transcription start sites, were included. In the case of *ZmCCT*, a CACTA transposon insertion that is known to play a role in photoperiod sensitivity was included in the design [22]. Primers were designed based on the B73 reference maize genome (AGPv3) using the Ion Ampliseq Designer (http://www.ampliseq.com) pipeline version 4.0. A nonstandard specificity, as opposed to a high or medium specificity design, as defined by the Ampliseq primer design algorithm, was used to increase the percentage of the region that was covered by the design (Table 1). These relaxed parameters may increase the possibility of off-target amplification. These primers were used on all samples and can be found in S1 File. The primers were split into two separate pools for the initial amplification, one pool with 160 amplicons, the other with 159 amplicons in order to improve amplification and sequencing results.

**Table 1:** Regions targeted by Ampliseq design. Targeted regions of interest are shown, along with the number of amplicons and coverage for each region.

Targeted region	Chromosome	Chromosome start	Chromosome end	Number of amplicons	Total targeted bases	Covered bases	Fraction of region covered
GRMZM2G154580	chr1	90221947	90224841	12	2894	2894	1
GRMZM2G011357	chr1	239667869	239673192	25	5323	5283	0.992
GRMZM2G180190	chr2	12649206	12654213	19	5007	4520	0.903
GRMZM2G095598	chr2	33216134	33219640	11	3506	2600	0.742
GRMZM2G033962	chr2	219433832	219441286	30	7454	6506	0.873
GRMZM2G031432	chr3	3986806	3988589	6	1783	1358	0.762
GRMZM2G031432	chr3	3990583	3990969	2	386	386	1
GRMZM2G031432	chr3	3994128	3996072	8	1944	1944	1
GRMZM2G031432	chr3	4139301	4140050	3	749	749	1
GRMZM2G045275	chr3	218979525	218987381	29	7856	6644	0.846
GRMZM2G067921	chr7	175583965	175587451	10	3486	2488	0.714
GRMZM2G179264	chr8	123030387	123034175	16	3788	3554	0.938
*vgt1*	chr8	131517263	131519147	10	1884	1868	0.992
GRMZM2G700665	chr8	131574889	131580316	16	5427	3902	0.719
GRMZM2G405368	chr9	35633308	35639846	23	6538	5354	0.819
GRMZM2G085218	chr9	106530026	106533123	11	3097	2641	0.853
GRMZM2G038783	chr9	108445974	108449794	13	3820	2964	0.776
GRMZM2G359322	chr9	123215070	123218079	9	3009	2012	0.669
GRMZM2G092174	chr9	135245567	135253882	34	8315	7302	0.878
GRMZM2G381691	chr10	94262291	94272461	32	10170	7228	0.711

### Library preparation

DNA was quantified using Picogreen (ThermoFisher, Grand Island, NY, USA). DNA from each sample was then normalized to 12.5 ng/uL. A total of 12.5 ng of gDNA, 1x primer pool and 1x master mix to a final volume of 10 uL was used for the initial amplification. For each sample, two initial amplification reactions were performed–one for each of two primer pools. The samples had an initial two-minute incubation at 99°C to activate the enzyme, followed by 19 amplification cycles of 99°C for 15 seconds, alternating with annealing steps of 4 minutes each. For cycles 1–3 an annealing temperature of 62°C was used, while for the remainder of the cycles an annealing temperature of 60°C was used. Pools were then combined, and 2 uL of 1x FuPa reagent added and incubated at 50°C for 20 minutes, followed by 55°C for 20 minutes and 60°C for 20 minutes to partially digest primer sequences.

Next, the barcodes and adapters were ligated onto the PCR products. A total of 95 samples were assayed. The diluted barcode adapter mix, FuPa product, 1x switch solution, and 2 uL of DNA ligase were then combined and incubated at 22°C for 30 minutes and 72°C for 10 minutes. To purify the libraries, 0.8 x magnetic beads (AMPure; Beckman Coulter Inc., Brea, CA, USA) were used, followed by two washes of 70% ethanol and 5 minutes of drying time. To equalize the concentration of the libraries and ensure that the same amount of DNA was included from each sample into the pooled sample, the Ion Equalizer Kit was used (Cat. 4482298; Thermo Fisher Scientific Inc.). A total of 50 uL of Platinum PCR SuperMix High Fidelity and 2 uL of Equalizer primers were added to the purified libraries. An amplification step at 98°C for 2 minutes and seven cycles at 98°C for 15 sec and 64°C for 1 minute were performed, and 10 uL of Equalizer Capture added. A total of 6 uL per reaction of washed Equalizer beads were used to equalize sample concentrations across the plate so that the same amount of DNA was included from each sample when libraries were pooled for a single sequencing run. Libraries were then pooled, emulsion PCR performed and sequenced using an Ion Torrent PGM 318 chip at the High-Throughput Sequencing Facility at the University of North Carolina-Chapel Hill. Sequencing reads have been deposited in the NCBI Sequence Read Archive and are available under SRA504653.

### Bioinformatics

In order to assess different mapping algorithms, we simulated Ion Torrent read data with read numbers similar to that obtained from the actual sequencing. First, we extracted the targeted regions from the reference genome file (B73 AGPv3.27). In order to simulate the data, we used this FASTA file with CuReSim 1.2 [23]. We used CuReSim as it was designed to simulate Torrent reads and because the error types generated by the simulated reads should be similar to that which we obtained in our actual dataset. We simulated reads that were of 191 bp in length and approximately ~62,000 reads. Two different methods were tested to map simulated reads: BWA-MEM version 0.7.13-r1126 [24] and Bowtie2 version 2.2.6 [25], with both software packages mapping the simulated reads to the B73 reference genome. Software versions and commands can be found in S2 Table.

For the actual dataset, Ion Torrent Suite software version 4.4.3 (Thermo Fisher Scientific Inc.) was used to filter and parse read data according to barcodes. BWA-MEM was used to map reads to the B73 RefGenv3 reference genome (AGPv3.27) [7] using default settings, as shown in S2 Table. Bowtie2 was also used to map reads to the reference genome using the ‘sensitive local’ setting, which has the following parameters: -D 15 -R 2 -N 0 -L 20 -i S,1,0.75 [25]. After reads were mapped and sorted using SAMtools version 1.3 [26], alignments were assessed using the CollectTargetedPcrMetrics function of picard tools version 1.136 (http://broadinstitute.github.io/picard) and depth of coverage using the DepthofCoverage function in GATK version 3.5 [27].

BWA-MEM alignments were used for variant calling on samples with more than 20,000 reads. The Genome Analysis ToolKit (GATK) version 3.5 was used to call variants [27]. Local realignment was performed using the RealignerTargetCreator and IndelRealigner functions in GATK. PCR duplicates were not removed as we expect that duplicates would be present due to the nature of PCR-based enrichment. Variants were called with HaplotypeCaller using GATK with a stand_call_conf value of 2.0 and a stand_emit_conf of 1.0. Resulting.g.vcf files were combined with the GenotypeGVCFs function of GATK. Variants were kept that fulfilled the following criteria: quality score greater than 30, quality by depth score greater than 5, and depth of coverage at a given genotype greater than 12. To compare variant calling methods, the SAMtools mpileup function was used to call variants with a maximum depth of 1000 using the GATK Indel Realigner alignments. SAMtools variants were then filtered so that only variants with higher than a 30 quality score and an individual genotype depth of 12 were included (under binomial sampling, this gives a 99.7% chance of sequencing either of the homologous chromosomes in an individual at least twice). Both datasets were filtered to remove indels. Indels are the most common Ion sequencing error, as the main error found in Ion data is inaccurate flow calls, or under-calling of long-homopolymers or over-calling of short-homopolymers [28]. We filtered out variants with an excess of heterozygotes and amplicons with a high proportion of variants with an excess of heterozygotes. First a *p*-value for excess heterozygosity was calculated for each variant using vcftools—hardy [29, 30]. Variants with a Bonferroni-corrected *p*-value less than 0.01 for an excess of heterozygotes were removed from the dataset. Additionally, all variants on amplicons where greater than 15% of variants were removed by the excess heterozygote filter were also filtered from the dataset.

To examine concordance between variant calling methods and between Ampliseq and previously reported whole genome sequence-based SNP calls, we filtered all called variants including the GATK and SAMtools variant datasets, as well as the maize HapMap3 dataset [31], to include only the regions that were within the designed intervals using the intersect function in BEDtools2 [32]. The vcf-compare function of vcftools version 0.1.14 was then used to compare resulting variant files [29]. Snpeff was used to annotate variants and predict their effect using the AGPv3.27 database [33].

We estimated the precision and sensitivity of heterozygous genotype calls following the usual definition of these terms in the classification literature [34] and assuming that sites at which parents were polymorphic correspond to F_1_ genotypes that are true heterozygotes. The sensitivity of heterozygous calls was estimated as the proportion of sites for which parents were polymorphic (true heterozygotes) that were scored as heterozygotes. The precision (or positive predictive value) of heterozygous calls was estimated as the proportion of heterozygous sites in the F_1_ hybrid controls at which the parents were polymorphic (i.e., the proportion of true heterozygotes among called heterozygotes). Multidimensional scaling was completed using PLINK [35]. F_ST_ [36] and total gene diversity were estimated based on 960 sites remaining after filtering out markers with more than 50% missing calls using the R package hierfstat [37]

## Results and Discussion

### Library preparation

A total of 95 diverse maize samples was used to evaluate PCR-based enrichment followed by semiconductor sequencing. This included the inbred lines B73, Mo17, and CML322 and the F_1_ hybrids B73×Mo17 and B73×CML322. These inbred lines were chosen because they are part of the HapMap3 dataset and have extensive genotypic data available from whole genome sequencing efforts [5, 31]. Hybrids were included to assess the ability of the method to genotype heterozygous individuals accurately. In addition to the control lines, five plants from each of 19 maize landrace accessions (expected to be non-inbred, highly heterozygous, and genetically variable [38]) were included to assess the ability of Ampliseq to amplify and genotype diverse maize samples. We expect there is some level of ascertainment bias for loci with the same sequences at the priming sites in this study, as we only used the B73 reference to design primers.

To test Ampliseq, we focused on 20 regions of the genome encompassing a total of 86 kb and containing candidate genes for photoperiod response in maize (Table 1). Candidate regions were selected based on previous knowledge of photoperiod response in maize (Table 1) [20–22]. The Ampliseq design was based on the B73 genome (AGPv3). A number of primer pool designs were created by the Ion Ampliseq assay design software, and a more relaxed design was chosen as it covered a greater percentage of the region of interest. A total of 72,197 bp of the 86,436 bp region was amplified by the design. On average, target candidate regions were 4.3 kb, of which an average of 3.6 kb was covered by the design. Genic regions were covered better than upstream and downstream regions. Some regions were not covered by the design, including GC- or AT-rich regions, regions within or near a repetitive sequence, or highly variable regions. Missed intervals were up to 76% GC, while other missed regions were as low as 27% GC (73% AT). The average amplicon size was 263 bp (range: 83–339 bp). On a region-by-region basis, coverage ranged from 71–100% (Table 1). Overall, the design covered 83.5% of the desired intervals.

**Fig 1:**
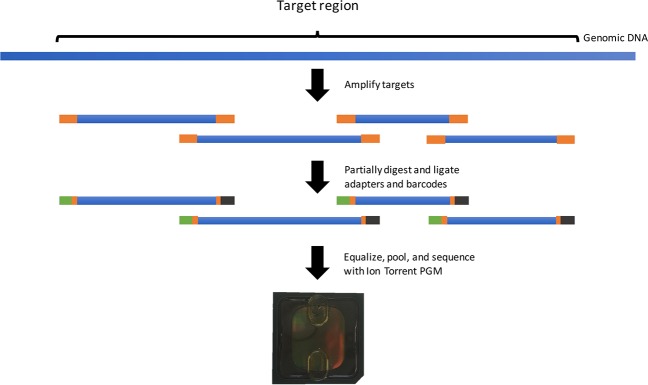
Ampliseq workflow. Target regions are selected and amplicons are designed to cover the region. Amplicons are then partially digested, and adapters and barcodes are ligated onto amplicons. Samples are then equalized and pooled. Sequencing was completed on an Ion Torrent PGM™.

The basic overview of the Ampliseq workflow is shown in Fig 1. Briefly, 12.5 uL of genomic DNA was used to amplify two primer pools targeting amplicons in the regions of interest, such that a total of 25 ng of DNA for each sample was needed. To improve amplification, primers were split into two separate pools by the Ampliseq Assay design software for the initial amplification stage. Following the purification step, amplicons were barcoded, and products purified and equalized. A total of 95 samples were then pooled together and sequenced.

### Semiconductor sequencing

We obtained a total of 6,405,140 high-quality reads with a total of 6,071,762 high-quality barcoded reads. The mean read length was 191 bp. Of the 95 samples submitted, we received more than 20,000 reads for 93 of the 95 samples. Thus, two samples did not have adequate sequencing reads to include in further analyses. A total of 98% of the reads mapped to the B73 reference genome. A total of 80% of the reads mapped to the targeted regions (Table 2).

**Table 2:** Simulations and alignments. The first two columns show the alignment statistics for the Bowtie2 and BWA-MEM alignments of the simulated data. The third column shows the alignment statistics for the actual data. For the actual data, the alignment statistics were averaged across all samples so that the per sample average is shown in the table.

	Bowtie2 alignment- simulated data	BWA-MEM alignment- simulated data	BWA-MEM alignment—actual data (average per sample)
Region of interest (bp)	74602	74602	74602
Total reads per sample	62301	62301	62301
Number of sequenced bases per sample	11587806	11587806	12064656
Percent of reads mapping to reference^1^	99	99	98
Percent of reads off-target	4.1	0.0	20
Mean amplicon coverage (reads per basepair)	148	147	105
Percent of bases at 2X	89	89	76
Percent of bases at 10X	84	85	66
Percent of bases at 12X	84	84	65
Percent of bases at 20X	81	82	60
Percent of bases at 30X	80	80	55

^1^For simulated data, this corresponds to the false negative rate.

### Alignments and simulation

**Fig 2:**
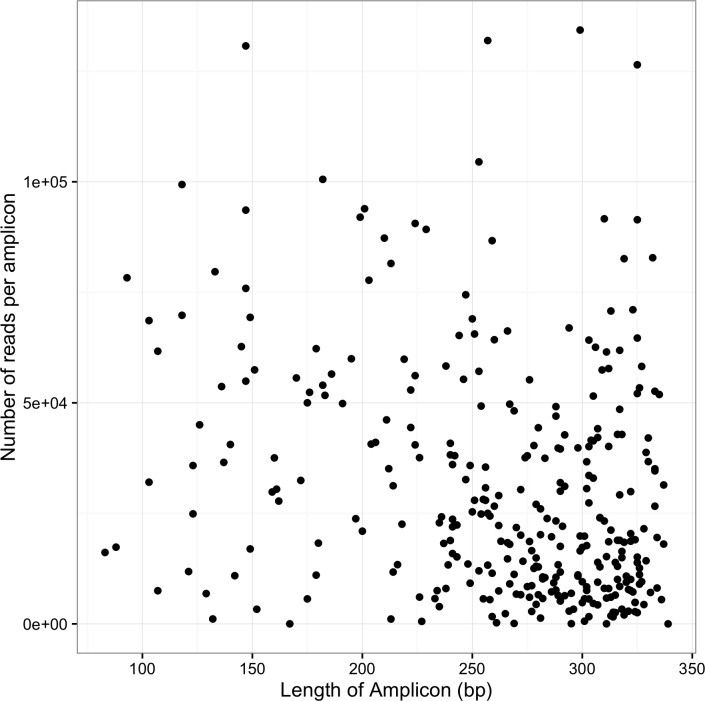
Number of reads per amplicon versus amplicon length. The number of reads per amplicon has little relationship with amplicon length. Long amplicons are represented.

In order to assess the quality of alignments obtained from different algorithms, we simulated Ion Torrent reads for the targeted region using CuReSim [23]. A total of ~62,300 reads were simulated, comparable to the number of reads generated for each sample by sequencing the library. Simulated reads were mapped to the B73 reference genome using BWA-MEM and Bowtie2, which allowed us to compare the different mapping tools (Table 2). The simulation results were used to guide us on choosing a mapping algorithm run under default settings. Our evaluation did not explore the alignment algorithm parameter space beyond the default values; previous studies have addressed alignment algorithm parameter choice [23, 39]. When comparing BWA-MEM with Bowtie2, we found that there were more off-target bases from the simulated reads with the Bowtie2 alignment (4.2%) than with BWA-MEM (0.0%). Because of the lower number of off-target alignments, we relied on BWA-MEM for mapping. Using BWA-MEM, approximately 98% of quality trimmed reads mapped to the B73 reference genome. On average across all samples, there was 105X coverage. It would be possible to increase the number of samples or bases sequenced on an Ion Proton 318 chip and still have adequate sequencing depth. One concern with PCR-based enrichment is a preference for shorter amplicons. We observed a lower depth of coverage for some longer amplicons; however, longer amplicons were still represented in the sequence library and little relationship was observed between the number of reads per amplicon and amplicon length (Fig 2).

When comparing the simulated data to the actual data, we found that there was off-target amplification with Ampliseq. That is, there was a higher rate of off-target mapping in the actual data (20% on average across all samples) than the simulated data (0.0–4.2%) (Table 2). The off-target sequences aligned to both genic and non-genic regions. Some of the regions that did not amplify are known to be regions with structural variation. For example, the region upstream of *ZmCCT* did not amplify in some samples. This region is known to harbor an insertion-deletion polymorphism that underlies variation in the response to photoperiod [22]. Lines that were known to lack the insertion had missing amplicons in the region, confirming that some missing amplicons are due to structural variation. When we selected the design, we used less stringent parameter settings than the default in order to cover a greater percentage of the intervals of interest. A more stringent design that covered a smaller percentage of the intervals may be a better choice when less off-target amplification can be tolerated. Because of the off-target amplification, we filtered variants and included only those in regions of the genome which were part of the Ampliseq primer design in downstream analyses.

Averaged across samples, 65% of the targeted bases were covered at greater than 12X. This corresponds to a 22% missing rate. This is lower than what may be expected given the high mean depth of coverage (105X), perhaps because of PCR bias or structural variation. The average percentage of targeted bases with zero coverage per sample was 17%. However, across all samples only 3.2% of targets had zero coverage. There is more than one reason that some amplicons may not have amplified in some samples, such as variation in the priming site, or presence-absence variation of the entire amplicon. Maize is known to harbor substantial amounts of sequence variation [31, 40], but additional experiments are needed to determine whether these amplicons are missing for this reason.

### Evaluation of variant calling methods

Previous work has shown that different variant calling programs result in non-identical variant datasets [41, 42]. Given our results from simulated sequence data (Table 2) we used BWA-MEM for mapping and tested both GATK and SAMtools mpileup to call variants. To compare different methods of variant calling, we examined only SNPs because the HapMapv3 dataset used in our comparisons included fewer indels with a different size distribution than our GATK and SAMtools datasets.

First, as a measure of the potential error rate of SNP calling, we examined the number of alternate alleles present in our sample of B73 relative to the reference genome sequence of B73 across the target space. Using GATK, based on two separate samples of B73, a total of 3 SNPs were called as homozygous for an alternative allele compared to the B73 reference sequence. Using SAMtools, 4 SNPs were called as homozygous alternate alleles. These differences may be due to methodological (algorithmic) errors or may be biological in nature. There may be some genetic variation between the B73 used for reference sequencing and the B73 line used in this study (our B73 sample and the HapMap3 sample were also not identical). Nevertheless, both algorithms provided nearly perfect calling accuracy.

To compare the robustness of the two variant calling methods with regards to heterozygous calls, we compared the genotypes of inbred lines B73, CML322, Mo17 to their F_1_ hybrids B73xCML322 and B73xMo17. The sensitivity of heterozygous calls was estimated as the proportion of heterozygous F_1_ calls among all cases when the parents were polymorphic. Heterozygous sensitivity was 80.8% for SAMtools and 96.5% for GATK. We also calculated the precision of heterozygote calling as the proportion of F_1_ heterozygous calls for which the parents were polymorphic. Heterozygous precision was 82.5% for SAMtools and 80.0% for GATK. Most of the false heterozygous calls were associated with sites at which the non-B73 parent line itself was scored as heterozygous; these sites were clustered in two genomic regions. We postulated that there may be additional paralogs in the non-reference line that were amplified and both the target gene and the reads from the paralog were aligning to the target gene region in B73, resulting in heterozygous calls in both the inbred parent and the F_1_. Therefore, we incorporated an additional filter to remove variant calls with an excess of heterozygotes. Since there was clustering of the heterozygous calls by genomic position, we also removed all variants on amplicons where greater than 15% of the calls failed the heterozygote filter. Applying these filters improved the precision of heterozygote calls to 89.5% for SAMtools and 92.5% for GATK without changing the specificity. Thus, we report a heterozygote accuracy rate of at least 80.8% for SAMtools and 92.5% for GATK.

Aside from bad alignments, there are other possible reasons for the erroneous calls. Since the same inbred line source was not used to generate the hybrid as was sampled for the inbred DNA, it is possible that there are some small differences between the inbred lines genotyped and those used to make the hybrids. That is, heterogeneity within the inbred lines could cause the genotyping to seem less accurate. In any case, the accuracy rates were higher for both measures in the GATK dataset, indicating that, under the parameter settings used, GATK is the better choice for variant calling of heterozygous materials.

**Fig 3:**
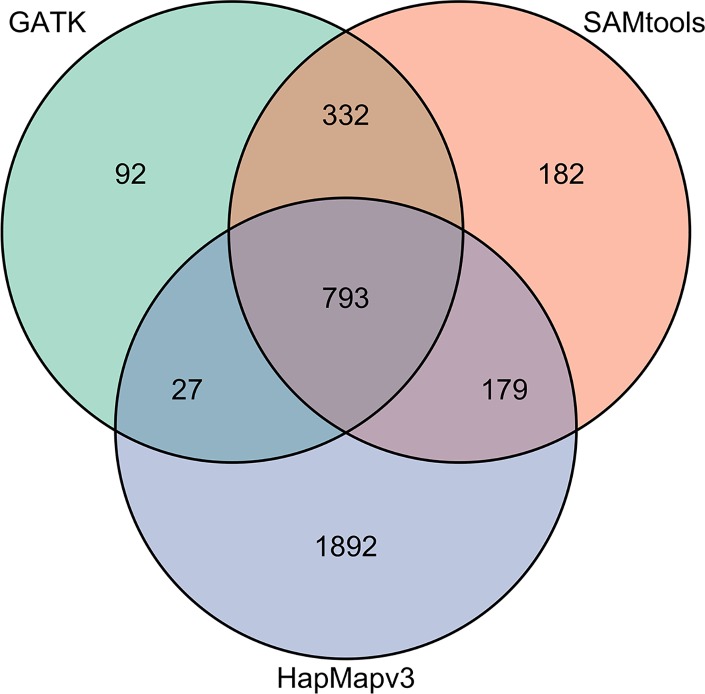
Venn diagram comparing different SNP datasets. Venn diagram representing the relationships among SNPs called using GATK and SAMtools, and the HapMap3 SNPs in the same genomic regions. Novel variants are those unique to the GATK and SAMtools datasets.

We also evaluated the reliability of the variant calling methods by comparing variants called in our dataset to the variants in the same regions in the HapMap3 dataset (Fig 3). A total of 1,605 high-confidence SNPs were identified using GATK or SAMtools across the 72,197 bp target space, corresponding to approximately 1 SNP per 45 bp. Among these SNPs, 70% were identified by both methods, while 7.4% and 22% were specific to GATK or SAMtools, respectively (Fig 3).

**Fig 4:**
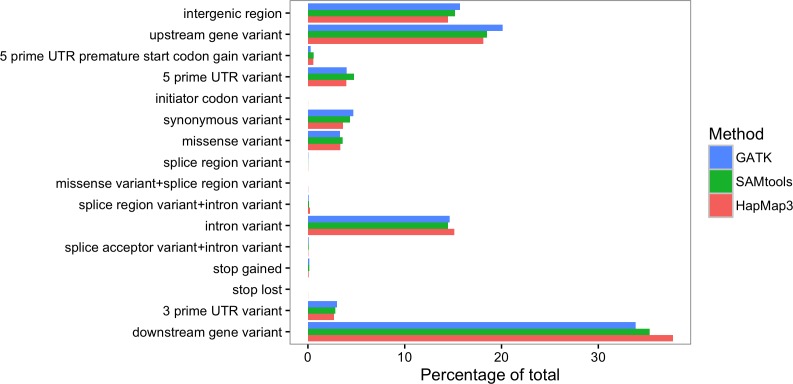
Variant classification by method. The distribution of variants classified according to genomic region or type of variation. GATK and SAMtools variant calls were compared to the HapMap3 variants in the region of interest.

HapMap3 included 2,891 SNPs across the target space, with 1,892 of these unique to HapMap3 and not found in our resequencing study samples. HapMap3 contains more SNPs in the targeted regions because SNP discovery was made in a broad and large sample of germplasm (916 lines) [31] while this study surveyed only three inbred lines and a small sample of 19 landraces from a limited geographic range. HapMap3 includes 42 inbred lines derived from 23 maize races and 19 wild teosinte relatives. The only race represented in the HapMap3 dataset that is also in our study was Cateto. Most SNPs in maize are rare [43], so our sample is expected to not include many of the rare alleles in the HapMap3 data set. In addition, SNPs in our germplasm sample may have been missed because of unrepresented amplicons or insufficient coverage across portions of the target space in some samples. For example, three of the amplicons had insufficient coverage across all samples to call variants, yet HapMap3 SNPs lie within those intervals. Also, the number of SNPs in the HapMap3 data is slightly inflated by errors in sequencing and variant calling, as the error rate of SNP calls in HapMap3 is estimated to be between 1–3% [31]. The SAMtools and HapMap3 datasets had 152 more SNPs in common than the intersection of GATK and HapMap3 (179 versus 27; Fig 3), indicating SAMtools was slightly more sensitive than GATK (overall, ~1.2X more SNPs were called by SAMtools versus GATK). In this study, we identified novel variants with a minimum allele frequency of 2% that were identified in at least two different samples. Stringent criteria were used to ensure that these variants are likely to be real. Among the 1,125 variants identified by both GATK and SAMtools, 30% have not previously been identified. This may be attributed to the germplasm sampled and the targeted sequencing approach where very high depth coverage was obtained at regions of interest. Although we only identified 35% of the total variants present in the same regions of the HapMap3 dataset, a high percentage (30%) of the variants were novel. The distribution of reads across types of annotations was similar between HapMap3, GATK variant calls and the SAMtools variant calls (Fig 4). The number of polymorphisms downstream of coding regions was a few percentage points higher in HapMap3 dataset.

**Fig 5:**
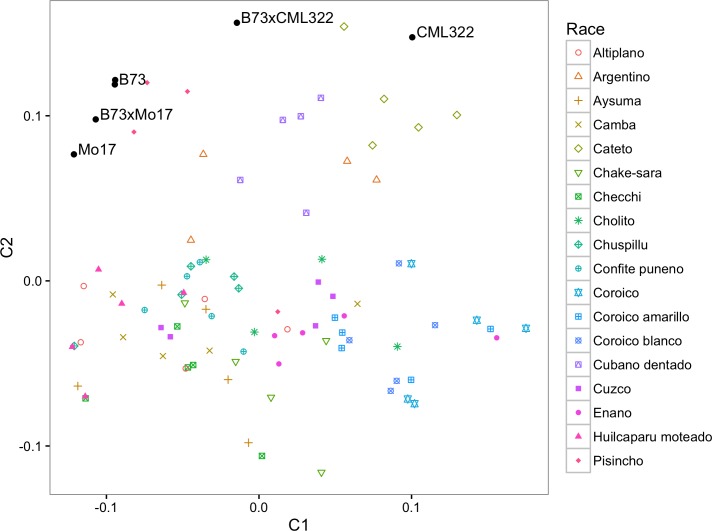
Multidimensional scaling applied to all samples at all resequenced loci. The plot includes five samples per accession and control samples.

Population stratification was observed in the materials included in this study. Overall gene diversity was estimated to be 0.26 and F_ST_ among accessions was estimated as 0.27, indicating substantial genetic variability and strong differentiation among the accessions. F_IS_ was estimated as 0.02, suggesting little inbreeding within populations overall, as expected for outcrossing populations. These results are very similar to diversity and F_ST_ estimates among maize accessions from Mexico based on isozyme data by Sánchez-González et al [38] and Doebley et al [44]. Patterns of relationships among the materials assayed visualized with multidimensional scaling (MDS) was consistent with historical and other genetic knowledge about the samples (Fig 5). As expected, the temperate inbred lines B73 and Mo17 grouped closely, with the hybrid halfway between the parents. B73 and Mo17 were most closely related to plants from the Argentine popcorn race *Pisincho* (ARG482), which is also the landrace accession furthest from the equator (-23°S). Also grouping more closely with the temperate inbreds was *Argentino*, a commercial, improved race [45]. The tropical inbred line CML322 grouped most closely with the Bolivian samples from the lowland races *Cateto* and *Cubano dentado*. *Cubano dentado* is similar to common yellow dents of the West Indies [45]. Historical descriptions of *Enano* and *Coroico* are congruent with the genetic evidence. The race *Enano* was separated from the *Coroico* races based on ear size, but it was suspected that they were closely related because the ears of both races had “enlarged bases to which the upper end of the shank adhered so strongly that it could be broken off only with difficulty” [45]. Indeed, in the MDS plot samples from these two races grouped closely, corroborating the historical documentation of these races with genetic data. Future experiments will more closely examine the relationships at these target genes among a larger sample of landrace accessions.

## Conclusions

Our results indicate that Ampliseq is a viable option to discover sequence variation and genotype heterozygous materials at the population scale in maize, which has a large, and complex genome. With the resequencing data, we were able to examine the genetic relationships between 19 landrace accessions from Bolivia and Argentina and found that the genetic data are congruent with historical descriptions of the relationships between races and indicated both high genetic diversity and strong population differentiation. A limitation of this approach is that limited structural variation will be revealed. In some cases, entire amplicons were missing for some samples, but further validation is needed to discern if this is due to structural variation. Another concern of this method is the off-target amplification for regions with high homology to other segments of the genome due to genome duplication. For this reason, we incorporated an excess heterozygosity filter and obtained a 96.5% sensitivity and 92.5% precision in calling heterozygotes using GATK. It may be advisable to use a more stringent design to reduce off-target amplification. This method is suitable for assaying a large sample. In this study, there was sufficient read depth when barcoding 95 samples and sequencing together in a single sequencing run to survey a total of nearly 72 kb across the genome. Based on the high depth of coverage we obtained in this study, it would be possible to increase the number of samples that are sequenced together to reduce costs, or maintain the PCR primer pair plex level and target two to five times more sequence space in a single sequencing run. The capacity to sequence more lines at a much lower cost per sample is a major advantage of this approach over whole genome sequencing. Advantages over other methods include the ability to multiplex many PCR primer pairs in a single reaction to obtain a high depth of sequence coverage at many loci, discover novel variation, and accurately score heterozygotes. The high coverage of targeted sites enables accurate calling of heterozygotes and this resequencing technique is useful when high coverage is needed for specific regions of interest. PCR-based sequence enrichment coupled with semiconductor sequencing is suitable to various applications, including marker-assisted selection, genetic mapping studies, and ecological studies.

## Supporting Information

S1 File Amplicon and primer information. Amplicon information and primer sequences used for amplification.
(CSV)

S1 Table Accession information. Race and collection information for resequenced landraces accessions.
(CSV)

S2 Table Software information. Information pertaining to the software, versions, and parameters used for data analysis.
(XLSX)
